# A novel self-assembled oligopeptide amphiphile for biomimetic mineralization of enamel

**DOI:** 10.1186/1472-6750-14-32

**Published:** 2014-04-27

**Authors:** Quan-Li Li, Tian-Yun Ning, Ying Cao, Wei-bo Zhang, May Lei Mei, Chun Hung Chu

**Affiliations:** 1 College & Hospital of Stomatology, Anhui Medical University, 230032 Hefei, China; 2 Faculty of Dentistry, the University of Hong Kong, 34 Hospital Road, Hong Kong, China

**Keywords:** Amelogenin, Peptide, Remineralization, Enamel, Biomimetic, Mineralization, Self-assemble

## Abstract

**Background:**

Researchers are looking for biomimetic mineralization of ena/mel to manage dental erosion. This study evaluated biomimetic mineralization of demineralized enamel induced by a synthetic and self-assembled oligopeptide amphiphile (OPA).

**Results:**

The results showed that the OPA self-assembled into nano-fibres in the presence of calcium ions and in neutral acidity. The OPA was alternately immersed in calcium chloride and sodium hypophosphate solutions to evaluate its property of mineralization. Transmission electron microscopy (TEM) and scanning electron microscopy (SEM) showed nucleation and growth of amorphous calcium phosphate along the self-assembled OPA nano-fibres when it was repetitively exposed to solutions with calcium and phosphate ions. Energy dispersive spectrometry (EDS) confirmed that these nano-particles contained calcium and phosphate. Furthermore, electron diffraction pattern suggested that the nano-particles precipitated on OPA nano-fibres were comparable to amorphous calcium phosphate. Acid-etched human enamel slices were incubated at 37°C in metastable calcium phosphate solution with the OPA for biomimetic mineralization. SEM and X-ray diffraction indicated that the OPA induced the formation of hydroxyapatite crystals in organized bundles on etched enamel. TEM micrographs revealed there were 20–30 nm nano-amorphous calcium phosphate precipitates in the biomimetic mineralizing solution. The particles were found separately bound to the oligopeptide fibres. Biomimetic mineralization with or without the oligopeptide increased demineralized enamel microhardness.

**Conclusions:**

A novel OPA was successfully fabricated, which fostered the biomimetic mineralization of demineralized enamel. It is one of the primary steps towards the design and construction of novel biomaterial for future clinical therapy of dental erosion.

## Background

Dental erosion is described as tooth surface loss produced by acids of non-bacterial origin and is now recognized as a major cause of tooth wear [1]. The treatment particular in advanced cases is complicated and difficult and is a great challenge to many dentists. Regeneration by biomimetic mineralization is a non-invasive therapeutic technique that has received increasing attention in the past decades [2]. A cell-free strategy of biomimetic mineralization has been proposed [2,3]. Researchers tried to mimic the natural process for biomineralization and developed inorganic–organic hybrid materials with a hierarchical microstructure that simulates the natural mineralized tissues [4]. This biomimetic mineralization method draws attention to the design of organic molecules as templates for controlling the nucleation and growth of mineral crystals and the design of the form of mineral-ion transport. It incorporates these two factors to form hierarchical hybrid materials under physiologic conditions [5,6]. Various in situ methods of biomimetic mineralization with promising results to regenerate the hierarchical microstructure of the enamel have been reported. The methods utilized gelatine gel loaded with calcium and phosphate ions [7], hydrogen peroxide containing calcium phosphate paste [8], hydrothermal method with control release of calcium from Ca-EDTA [9], surfactant assembly hydroxyapatite [10], self-assembly of amphiphilic dendrons [11], electrolytical deposition taking place at 85°C [12], and amelogenin [13].

Matured enamel is composed of 95 wt% nano-rod-like hydroxyapatite. It is crucial to design the macromolecules as the template to induce the enamel microstructure regeneration under physiological conditions [14]. During enamel formation, amelogenin, the major enamel protein constituting approximately 90% of all organic matrix material [15], spontaneously self-assembles into nano-spherical supermolecular structures. Amelogenin provides an effective template to induce enamel microstructure regeneration. However, it can be easily denatured and expensive to extract. Oligopeptides are less costly to prepare and more stable than natural protein. This biomaterial has been used as a molecular tool for the synthesis, growth, and assembly of nano-structured materials [16]. Hartgerink et al. developed a peptide amphiphile that contains an alkyl tail and an ionic oligopeptide [17]. The peptide amphiphile is able to self-assemble into nano-fibre networks. It can promote the mineralization of hydroxyapatite to form a composite material in which the crystallographic c-axes of hydroxyapatite are aligned with the long axes of the fibres. Kirkham et al. developed an oligomeric β-sheet-forming peptide that spontaneously undergoes hierarchical self-assembly into fibrillar scaffolds. It can induce hydroxyapatite nucleation de novo and facilitate remineralization of caries-like lesions [18].

Amelogenin spontaneously self-assembles into nano-spherical, supermolecular structures during enamel formation. Afterwards, it self-assembles into birefringent, microribbon structures [19]. This supermolecular structure plays a vital role in controlling enamel hydroxyapatite crystal co-assembly [19]. The molecular mechanism of amelogenin self-assembly involves the primary structure of the protein maintaining a bipolar nature, which leads to unique globular monomers with flexible, hydrophilic, C-terminal (-Thr-Lys-Arg-Glu-Glu-Val-Asp) “tails” on globular monomer surfaces [19]. The hydrophilic C-terminal “tail” interacts with calcium ions and initializes hydroxyapatite nucleation [20]. This study aimed to fabricate a self-assembly anionic oligopeptide amphiphile that contains the hydrophilic functional domain of amelogenin to initialize hydroxyapatite nucleation and promote biomimetic mineralization of demineralized enamel.

## Methods

### Oligopeptide amphiphile fabrication

The oligopeptide amphiphile (C_18_H_35_O-Thr-Lys-Arg-Glu-Glu-Val-Asp) fabricated in this study consists of “two parts” hydrophilic C-terminal amino residue of amelogenin (-Thr-Lys-Arg-Glu-Glu-Val-Asp) and a derivative of stearic acid (C_18_H_35_COOH) (Sigma-Aldrich, St. Louis, MO, USA). The amino residue of amelogenin was used as the hydrophilic functional group, and the stearic acid derivative was used as the hydrophobic tail. The oligopeptide amphiphile was synthesized using standard, Fmoc solid-phase, peptide synthesis (SPPS, Additional file 1: Figure S1), which proceeded step by step in a C-terminal to N-terminal fashion using automated synthesizers. The synthetic oligopeptide was purified by ammonium sulphate precipitation and reverse-phase, high-performance liquid chromatography (HPLC). It was characterized by high-performance liquid chromatography (HPLC) and mass spectrometry.

### Self-assembly of the oligopeptide induced by calcium ions

Oligopeptide solutions at concentrations of 1, 2.5, 5, or 10 mg/mL were prepared by dissolving the oligopeptide into 0.1 M sodium hydroxide solution (Sigma-Aldrich, St. Louis, MO, USA). The alkalinity of the oligopeptide solution was then adjusted to neutral (pH = 7.0) by adding hydrochloric acid. A 100 μL aliquot of a 1 M calcium chloride (CaCl_2_) solution (Sigma-Aldrich, St. Louis, MO, USA) was added to 1 mL of the oligopeptide solutions at concentrations of 1, 2.5, 5.0, or 10.0 mg/mL. A suspension was formed and mixed thoroughly in a vial after each drop of the CaCl_2_ solution to promote diffusion of the metal ions. The self-assembled oligopeptide was characterized by transmission electron microscopy (TEM) and scanning electron microscopy (SEM) as the following. A suspension of 10 mg/mL self-assembled oligopeptide was ultrasonically diluted with ethanol. The diluted suspension was smeared onto a carbon-coated TEM grid and examined under TEM (JEOL-2010, JEOL Ltd. Co., Tokyo, Japan). For SEM examination (Sirion 200, FEI Co., Hillsboro, OR, USA), the oligopeptide-coated TEM grids were fixed with 2% glutaraldehyde in phosphate-buffered saline (PBS) (pH = 7.4) at 23°C for 2 h. It was subsequently dehydrated in ethanol and critical-point dried before gold sputtering.

### Biomimetic mineralization property of the oligopeptide nano-fibres

Self-assembled oligopeptide was deposited onto a porous carbon-coated TEM grid and fixed with 2% glutaraldehyde in PBS (pH = 7.4) at 23°C for 2 h. Grids coated with the oligopeptide were first immersed in a 10 mM CaCl_2_ solution for 1 h at 23°C and then washed with deionized water. Afterwards, the samples were exposed to a 5 mM sodium hypophosphate (Na_2_HPO_4_) solution for 1 h at 23°C and washed with deionized water. The protocol of CaCl_2_ followed by Na_2_HPO_4_ immersion was repeated five times. The specimens were examined under TEM and SEM to study the biomimetic mineralization of the oligopeptide nano-fibres. Electron diffraction was used to study the crystal structure. Energy dispersive spectrometry (EDS) was used to evaluate the calcium and phosphorus content.

### Biomimetic mineralization of enamel in metastable calcium phosphate solution

Sound extracted human molars were collected with ethics approval (IRB UW 10–210). Eight enamel slices of 1 mm in thickness were prepared perpendicular to the longitudinal axis of the tooth using a low-speed IsoMet diamond saw (Buehler Ltd., Lake Bluff, IL, USA). The slices were polished with 1200-grit silicon carbide paper under running water. The slices were checked under microscope (×10) for cracks before they were cleaned in ethoxylate detergent solution, acetone, ethanol, and deionized water. The enamel slices were demineralized by acid-etching with 37% phosphoric acid (H_3_PO_4_) for 60 s, rinsed with deionized water, and stored in a polyethylene tube at 4°C.

The metastable calcium phosphate solution was prepared as suggested by Fan [21]. It contained 2.58 mM calcium (CaCl_2_ · 2H_2_O), 1.55 mM phosphates (KH_2_PO_4_), 1 mg/L sodium fluoride (NaF), and 180 mM sodium chloride (NaCl). It was buffered to a pH of 7.6 by 50 mM of trihydroxymethylaminomethane (Tris)-hydrochloric acid (Sigma-Aldrich, St. Louis, MO, USA). The biomimetic mineralizing solution was developed by adding oligopeptide. It was added to the buffered metastable calcium phosphate solution to achieve a concentration of 15 μg/mL. In order to reveal the particle size distribution of Ca^2+^-induced self-assembled peptide, the biomimetic mineralizing solution was characterized at 25°C by a nano-particle size analyser (Zetasizer Nano S90, Malvern Instruments Ltd, UK). Particle size distribution was studied using multimodal algorithms based on the size distribution of the scattering light intensity. The stability of the biomimetic mineralizing solution and the metastable calcium phosphate solution were assessed by examination for precipitation after 30 days. The precipitate formed, if any, was studied using TEM.

Two tooth slices were placed into a polyethylene tube containing 15 mL biomimetic mineralizing solution and incubated at 37°C for 1 day. Another 2 slices were incubated for 20 days. The biomimetic mineralizing solution was refreshed every 2 days. The tooth slices after incubation were washed with deionized water and air-dried before examination under SEM and X-ray diffraction (XRD) (X’Pert Pro, Philips, Almelo, The Netherlands). A microhardness tester (Type M, Shimadzu Corp., Kyoto, Japan) was used to measure the Vicker’s hardness on the enamel of the tooth slices. The enamel surface was subjected to a load of 100 × 10^-3^N for 10 s at each test point for microhardness assessment. The above experiments were repeated on 4 tooth slices using the biomimetic mineralizing solution without the oligopeptide as a control.

The collected data was entered into an Excel file and checked to minimize data entry errors. The data was then analysed with statistical software SPSS 17.0 for Windows (SPSS Inc. Chicago, Illinois, USA). GLM univariate analysis will be used to check normality and variance of the samples. One-way analysis of variance and Tukey’s test were used to compare the microhardness of the enamel surface i) with or without H_3_PO_4_ etching enamel and ii) after incubation in biomimetic mineralizing solution with or without the oligopeptide. The level of significance was 5%.

## Results

### Oligopeptide amphiphile fabrication

**Figure 1:**
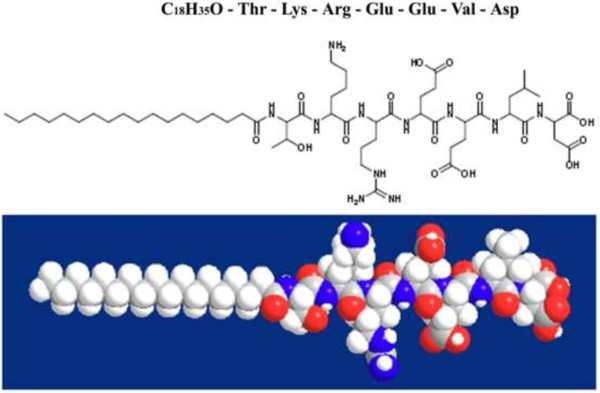
The chemical structure of the oligopeptide amphiphile.

**Figure 2:**
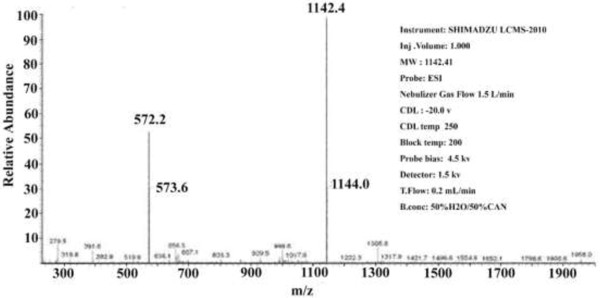
Mass spectrometry report of the oligopeptide.

**Figure 3:**
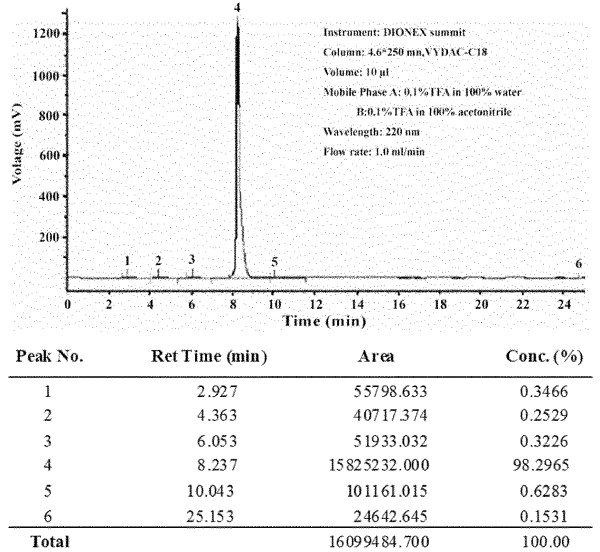
HPLC report of the purified oligopeptide.

The oligopeptide amphiphile consists of an anionic oligopeptide and an alkyl tail and its chemical structure was shown in Figure 1. The results of the mass spectrometry showed that molecular weight of the synthetic peptide was 1,142.4 (Figure 2), which corresponds to the designed oligopeptide amphiphile (C_18_H_35_O-Thr-Lys-Arg-Glu-Glu-Val-Asp). HPLC demonstrated that the purity of the synthetic peptide was 98.29% (Figure 3).

### Self-assembly of the oligopeptide induced by calcium ions

**Figure 4:**
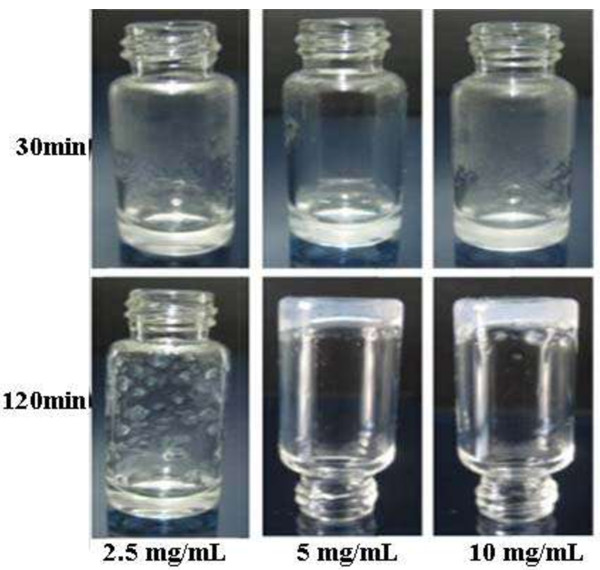
Effect of the oligopeptide solution after adding calcium chloride according to time and oligopeptide concentration.

The oligopeptide solution formed a hydrogel after adding calcium chloride. The gel strength and viscosity increased with time and oligopeptide concentration (Figure 4). A thick gel that could stick to the bottom of an inverted vial was formed at oligopeptide concentrations of 5 mg/mL and 10 mg/mL after 120 minutes.

### Biomimetic mineralization property of the oligopeptide nano-fibres

**Figure 5:**
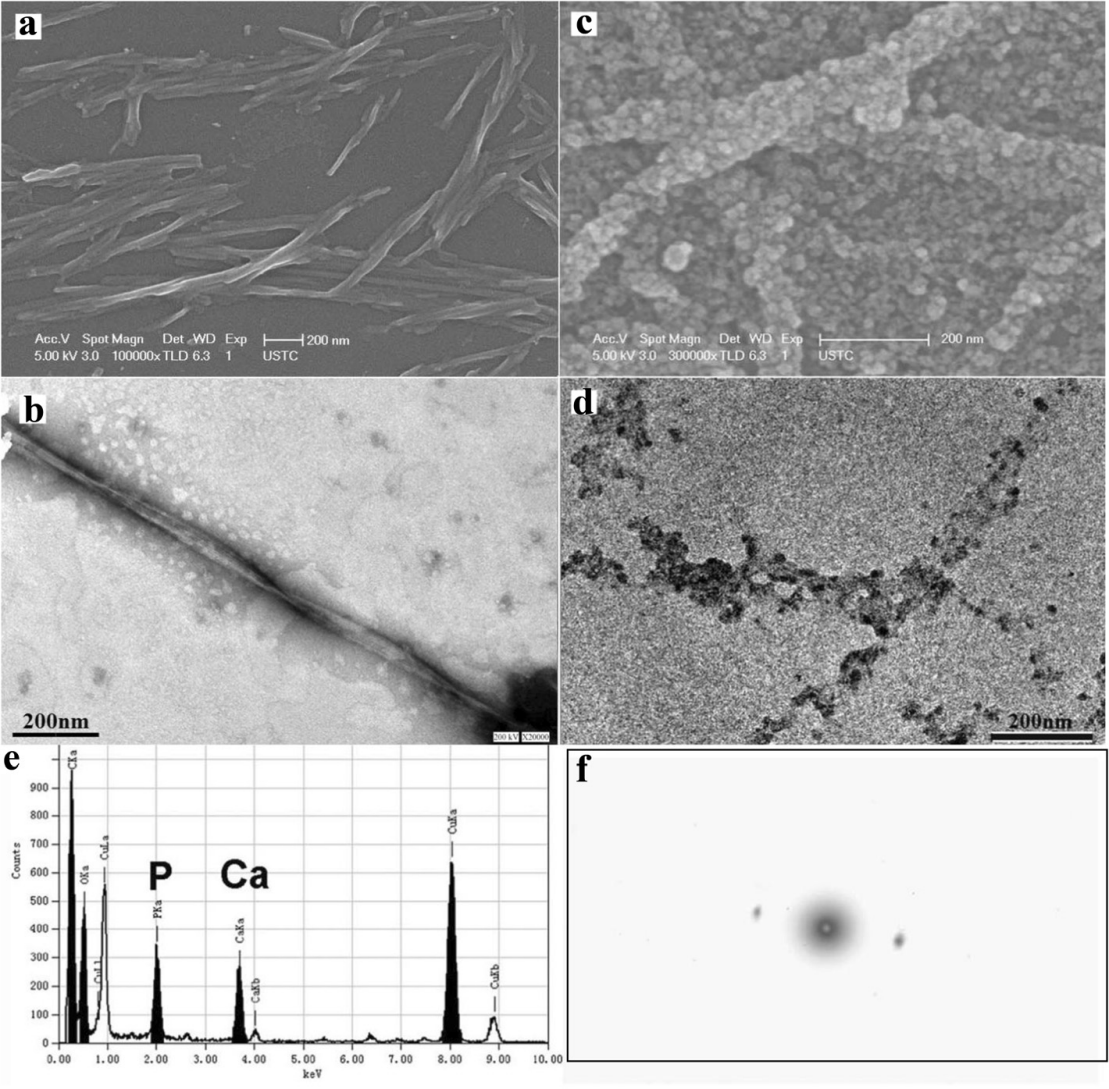
**Biomimetic mineralization property of the self-assembled oligopeptide. (a)** SEM and **(b)** TEM (negative stain with phosphotungstic acid) micrographs of the self-assembled oligopeptides. **(c)** SEM and **(d)** TEM (dense electronic images) micrograph showing precipitation of nanoparticles along the oligopeptide fibers. **(e)** EDS and **(f)** electron diffraction pattern of the precipitated nanoparticles.

SEM and TEM micrographs demonstrated that the self-assembled oligopeptide had well-defined nano-fibres with a clear helical twist (Figure 5a and b). The width of the fibres was approximately 12 nm, with a full-twist pitch of approximately 240 nm. The nano-fibres varied in length and the long fibre could be several micrometers. After the self-assembled oligopeptide was alternately immersed in a calcium ions solution and a phosphate ions solution for 5 cycles, abundant nano-particles were found precipitated along the oligopeptide fibres, forming a corn-on-the-cob appearance (Figure 5c and d). Results of EDS confirmed that these nano-particles contained calcium and phosphate (Figure 5e). There were few noticeable diffraction arcs in the resulting interference pattern, and the electron diffraction pattern suggested that the precipitates were comparable to amorphous calcium phosphate (ACP) (Figure 5f).

### Biomimetic mineralization of enamel in metastable calcium phosphate solution containing the peptide

**Figure 6:**
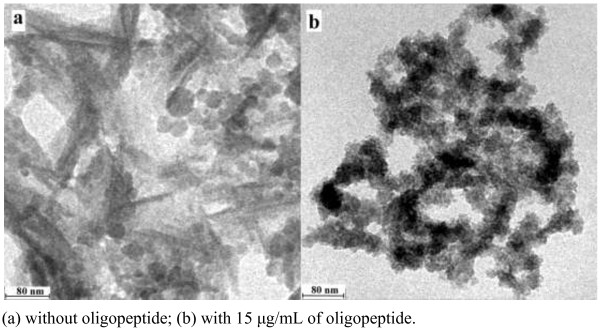
**TEM micrographs of the precipitates from the calcification solution that settled over 30 days at 37°C. (a)** without oligopeptide; **(b)** with 15 μg/mL of oligopeptide.

**Figure 7:**
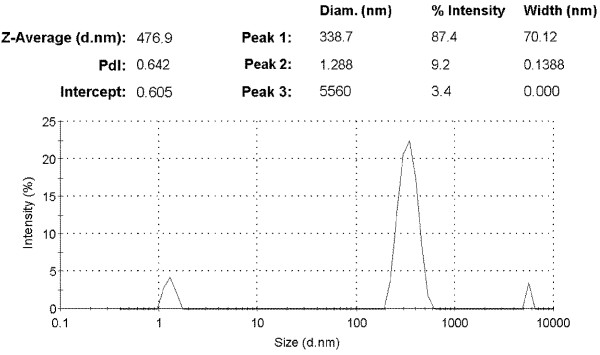
The particle size distribution in the metastable calcium phosphate solution with oligopeptide.

Flocculent precipitates were observed in the metastable calcium phosphate solution after 30 days. TEM micrographs revealed that precipitates contained ACP and apatite (Figure 6a). This is probably because ACP spheres were partly transformed into apatite crystallite clusters. The calcification solution with oligopeptide was relatively clear with few precipitates over a period of 30 days. TEM micrographs revealed that the precipitates were 20–30 nm nano-ACP particles (Figure 6b). The particles were found separately bound to the oligopeptide fibres. No apatite crystal was observed. The final concentration was 2.58 mM calcium, 1.55 mM phosphate, 1mg/L NaF, 180 mM NaCl, and 15 μg/mL oligopeptide, which was buffered by 50 mM trihydroxymethylaminomethane (Tris)-hydrochloric acid to a pH of 7.6. The particle sizes of the Ca^2+^-induced self-assembled peptide in the biomimetic mineralizing solution was characterized by a nano-particle size analyser (Figure 7). The size of the particles was mostly 338.7nm (87.4%). There are also two small peaks lying in 1.288 nm (9.2%) and 5,560 nm (3.4%), which may possibly be due to the presence of short oligopeptide nano-fibres and nano-fibres aggregation.

**Figure 8:**
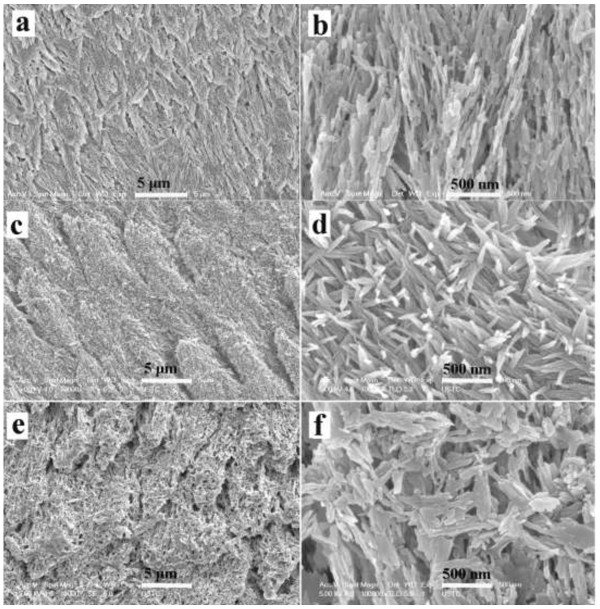
**SEM micrographs of enamel surface at different magnifications. (a, b)** Demineralized surface after acid etching. **(c, d)** Hydroxyapatites precipitated on the enamel surface after 24 hours without oligopeptide. **(e, f)** Hydroxyapatites precipitated on the enamel surface after 24 hours with oligopeptide.

**Figure 9:**
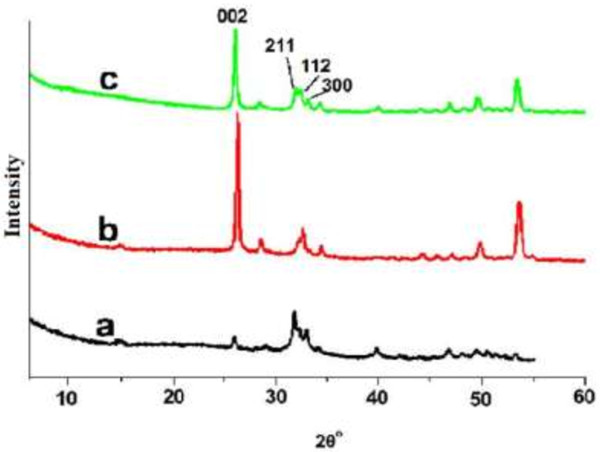
**XRD spectra of the specimens. (a)** Enamel after acid etching. **(b)** Precipitates on enamel without oligopeptide after 20 days**. (c)** Precipitates on enamel with oligopeptide after 20 days.

Figure 8 shows the precipitation of crystals on the enamel surface with or without oligopeptide after 24 h. XRD confirmed that the crystals were hydroxyapatite (Figure 9). When the partially demineralized enamel slices were incubated at 37°C in a metastable calcium phosphate solution (Figure 8a, b), hydroxyapatites were found precipitated in a homogeneous manner on the demineralized enamel surface. However, the crystal morphology on the enamel surface with metastable calcium phosphate solution contained no oligopeptide and was different from that with a metastable calcium phosphate solution with oligopeptide. SEM (10,000×) showed that hydroxyapatite crystals were uniformly precipitated on the pre-existing enamel crystals when there was no oligopeptide (Figure 8c). Higher magnification (100,000×) showed that they were needle-like, well defined, and precipitated in a relatively isolated and separate manner (Figure 8d). In the presence of the oligopeptide, the precipitated hydroxyapatite crystals were obtuse and rounded at both ends. These blunted, rod-like crystals were smaller in size than those crystals grown without oligopeptide (Figure 8e, f).

**Figure 10:**
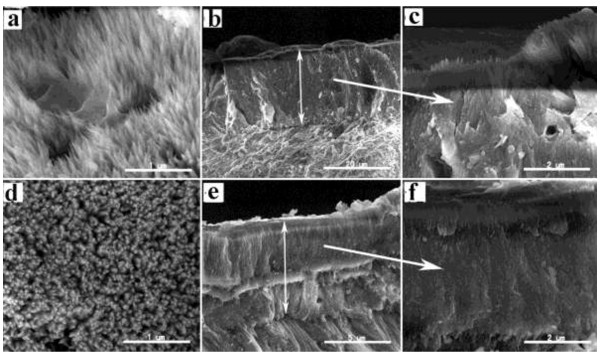
**SEM micrographs of hydroxyapatites precipitated on enamel surface. (a)** Precipitates without oligopeptide. **(b)** Transverse section of **(a)**. **(c)** High magnification of **(b)**. **(d)** Precipitates with oligopeptide. **(e)** Transverse section of **(d)**. **(f)** High magnification of **(e)**.

The morphology of the precipitated hydroxyapatite crystals after 20 days is shown in Figure 10. The precipitates in the absence of the oligopeptide were spiky, needle-like hydroxyapatite crystals (Figure 10a, b); those precipitated in the presence of the oligopeptide were rod-like hydroxyapatite crystals and tended to be more bundled (Figure 10d, e). The cross-section of the enamel slices showed that homogeneous hydroxyapatite crystals were oriented in parallel and packed together to form a dense, uniform layer. The thickness of the precipitated crystal layer in the presence of the oligopeptide was approximately half of that without oligopeptide (Figure 10b, e). The rod-like precipitated crystals with oligopeptide were more densely packed than those spiky crystals precipitated in the absence of oligopeptide.

Figure 9a showed XRD spectra of enamel samples. The diffraction peaks (002), (211), (112), and (300) planes corresponded well to the peaks for hydroxyapatite. The ratios of diffraction intensity of the c-axis (002) reflection to the diffraction intensity of the (211) or a-axis (300) reflections were considerably enhanced in all the calcium phosphate-coated samples compared to the etched tooth surface. The result suggested that the precipitated hydroxyapatite was oriented along its c-axis. The size of the precipitate crystal along the c-axis (length) and a-axis (width) was calculated using the Debye-Scherrer formula, D = 0.89λ/(β1/2 * cosθ) (D is the crystallite size; λ is the wavelength of the X-rays; and β1/2 is the diffractometry (XRD) pattern broadening). The length of the crystal was shorter in the presence of the oligopeptide than that formed without the oligopeptide. The width of the crystal was affected to a lesser extent. The results match the observations from the SEM (Figure 10).

**Figure 11:**
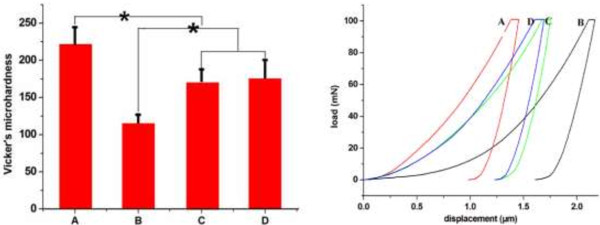
**Vicker’s hardness of the enamel specimens.** A - untreated enamel, B - demineralized enamel by acid-etching, C - enamel coated with hydroxyapatite precipitates without the oligopeptide, D - enamel coated with hydroxyapatite precipitates in the presence of the oligopeptide. * p < 0.05. The right pane is the typical load–displacement curve of different samples under the Vicker’s microhardness test.

The mean values of Vicker’s hardness of (A) untreated enamel, (B) demineralized enamel by acid-etching, (C) enamel coated with hydroxyapatite precipitates without the oligopeptide, and (D) enamel coated with hydroxyapatite precipitates in the presence of the oligopeptide are shown in Figure 11. The results showed a statistically significant difference among the 4 groups. A has the highest mean value of Vicker’s hardness, whereas B has the lowest. The results showed that acid-etching significantly reduces enamel microhardness. Biomimetic mineralization with or without the oligopeptide increased demineralized enamel microhardness. However, the microhardness is still lower than that of the untreated enamel. There was no significant difference in hardness between the remineralization groups regardless of oligopeptide use.

## Discussion

The oligopeptide used in this study is fabricated by SPPS, one the most common techniques for peptide synthesis, which proceeded step by step in a C-terminal to N-terminal fashion and ended with the alkylation of the NH_2_ terminus of the peptide [22]. HPLC and mass spectrometry were used to characterize and evaluate the purity of the oligopeptide [23]. This study demonstrated that it can be synthesized and purified to a high degree of purity although it is a relatively long molecule. The synthetic oligopeptide is amphiphilic because it has a hydrophilic anionic oligopeptide with a hydrophobic tail. The stearic acid derivative has a long carbon alkyl chain which was used as the hydrophobic tail. It was reported that the alkyl tail attached to the NH_2_ or COOH termini of peptide could influence their aggregation and secondary structure in water in both synthetic and natural systems [17]. The hydrophobic alkyl tails would pack together in the centre and leave the peptide segments exposed to the aqueous environment, resulting in peptide amphiphile self-assembly. The amino acid sequence of the peptide derived from the hydrophilic C-terminal amino residue of amelogenin and acts as a functional domain to induce mineralization. The oligopeptide can be dissolved in an alkaline solution but is insoluble in neutral or acidic solutions, which may contribute to its hydrophilic anionic peptide sequence. The results suggested that calcium ions induced the oligopeptide to self-assemble. It is reported that it is an essential step of supermolecular assembly for the oligopeptide to act function [18,19]. We considered that the property of calcium ions’ induced self-assembly may facilitate the mineralization to capture and trap calcium ions within the mineralized areas. Although the results cannot confirm that the self-assembly oligopeptide is able to capture calcium and phosphate ions from the solutions and to initiate the nucleation, this study demonstrated the peptide can induce ACP to form along the self-assembly fibres when it is alternately immersed in a calcium ions solution and a phosphate ions solution. Quantitative assessment of the calcium and phosphate content of ACP such as atomic absorption spectroscopy is essential, but the quantity of mineralized peptide fibres was too little for assessment.

The biomineralization process is an organic matrix-mediated mineralization other than a simple thermodynamic and kinetic process of inorganic mineral ions crystallization under aqueous solution condition [24]. A key aspect of this organic matrix-mediated deposition concerns the role of molecular interactions in controlling oriented nucleation at the matrix-mineral interface. In this study, the flocculent precipitates containing ACP and apatite were observed in the metastable calcium phosphate solution without oligopeptide addition after 30 days. This is probably because ACP spheres were partly transformed into apatite crystallite clusters. However, the calcification solution with oligopeptide was relatively clear with few precipitates of 20–30 nm nano-ACP particles over 30 days. No apatite crystal was observed. This suggests that the nano-ACP precursor is obtained by adding the oligopeptide to metastable calcium phosphate solutions with fluoride. The negatively charged peptide binds to ACP nano-precursors and sequesters the ions. There is inhibition of crystal nucleation and induction of liquid-liquid-phase separation in the aqueous crystallizing solution [25,26]. The sequestered ACP presents as droplets that appear transparent to the naked eye because they are smaller than the wavelength of light. Because of the sequestering mechanism, the oligopeptide-ACP complex can prevent ACP nano-precursors from aggregation and precipitation and make them small. Therefore, the nano-ACP precursor is highly active and can be transported to the mineralizing zone to induce mineralization [27].

More and more evidence supports the notion that biomineralization is a non-classical crystallization pathway and a bottom-up approach [5]. In such cases, crystallization often proceeds by a sequential process involving structural and compositional modifications of amorphous precursors and crystalline intermediates. Crystallization often involves an initial amorphous phase (such as ACP) that may be non-stoichiometric, hydrated, and susceptible to rapid phase transformation [6]. According to this concept, a biomimetic mineralization method has been successfully utilized in the duplication of a hard tissue structure [28]. Acidic macromolecules are added to a metastable calcium phosphate solution to stabilize the amorphous mineral phases and to reduce these amorphous phases to the nanoscale [29]. Crystal growth is then controlled by the macromolecules that undergo self-assembly. In this study, we examined the initial nucleation and growth (24 h) of the precipitated crystals and the speed of HA crystal growth (20 days) induced by the oligopeptide. It is more desirable to study the samples in multiple time point. It is our primary study and communication report, and more time point should be studied to explain the HA crystal growth in the following study.

In this study, the microhardness of the samples with hydroxyapatite precipitates in the presence of oligopeptide was similar to those without oligopeptide. Homogeneous hydroxyapatite crystals were oriented in parallel and packed together to form a dense, uniform layer. As the crystals on the surface were newly mineralized, they did not reach the mature stage of the crystal that formed underneath. This agrees with the findings reported by Busch [7] that there was no significant difference in hardness between the remineralization groups regardless of oligopeptide use.

Primary and secondary biomimetic growth of calcium phosphate can be distinguished on polymer surfaces. It is however not easy to distinguish the growth with the SEMs provided in this study. Mature enamel hydroxyapatite crystals bundled in parallel to form a special microstructure of repeating units of prisms. It is plausible that the structure of the precipitate was similar to the microstructure of the enamel. It is better to evaluate the remineralization with a quantitative method. However, the precipitate and the enamel had similar composite and structure; even the atomic absorption spectroscopy could not show the difference.

This study did not assess the stability or incorporation of the hydroxyapatite precipitates onto the enamel surface. In addition, the thickness of the crystal layer precipitated in the presence of the oligopeptide was less than that formed without oligopeptide. Therefore, the presence of the oligopeptide reduced the precipitation rate of hydroxyapatite crystal. It is noteworthy that the precipitated crystals with oligopeptide were more densely packed than those precipitated without oligopeptide. This is probably due to the different rate of crystal precipitation. The results showed that the morphology and the growth of the precipitated layer (remineralized layer) was different between the experimental samples (with the oligopeptide) and the control samples (without oligopeptide). Compared to the control samples, the advantage of using the oligopeptide is that the crystals are more densely packed. The disadvantage was that the deposition rate is slow in the presence of oligopeptide. However, the slow speed of crystal growth contributes to crystals packing densely. The metastable calcium phosphate solution with oligopeptide is more stable than the metastable calcium phosphate solution without oligopeptide. This advantage will facilitate its potential clinic use.

Bertassoni and his co-workers proposed that a more appropriate endpoint to evaluate the effectiveness of remineralization should be associated with the recovery of the mechanical properties of the hydrated dental tissue, which is presumed to correlate well with its overall functionality [30]. Hardness testing is an indirect method of tracking changes in the mineral content of dentin [30]. Biomechanical properties of dental tissues in general have been mostly evaluated by microhardness testing methods [31]. A disadvantage in microhardness assessment is the lack of analysis of reading error [32]. Although reading errors may have occurred in this study, the findings of microhardness measurement are consistent with the SEM and TEM observations. In addition, any reading error in this investigation should be small because the rater is experienced in microhardness testing and became fully acquainted with the procedure.

## Conclusion

A novel self-assembly oligopeptide amphiphile was fabricated with high purity. The oligopeptide self-assembles into nano-fibres in the presence of calcium ions and at neutral acidity. The self-assembled oligopeptide allows precipitation of ACP along its nano-fibres when it was alternately immersed in a calcium ions solution and a phosphate ions solution. The metastable calcium phosphate solution with the oligopeptide can induce formation of a relatively stable nano-ACP complex precursor. The nano-ACP complex precursor can induce nano-rod hydroxyapatite growth which densely packed and bundled together to form enamel-like tissue on demineralized enamel and the precipitation of these crystals increases enamel hardness. This synthetic novel oligopeptide can be a potential molecular tool for biomimetic mineralization on the enamel surface. It is one of the primary steps towards the design and construction of novel biomaterial for future clinical care of dental erosion.

## Competing interests

The authors declare that they have no competing interests.

## Authors’ contributions

QLL and CHC designed the oligopeptide amphiphile, supervised the project, and prepared the manuscript. WL was involved in the SPPS, HPLC, and mass spectrometry. YC prepared the tooth slides and oligopeptide solutions and performed SEM. TYN helped in all procedures, performed TEM and data analysis. All authors read and approved the final manuscript.

## Supplementary Material

Additional file 1: Figure S1 Fmoc SPPS solid-phase, peptide synthesis. Click here for file
