# Supplementary material for: A novel self-assembled oligopeptide amphiphile for biomimetic mineralization of enamel
Source: BMC Biotechnol. 2014 Apr 27;14:32. doi: 10.1186/1472-6750-14-32 (PMC4021083; doi:10.1186/1472-6750-14-32)

Additional file 1: Fmoc SPPS solid-phase, peptide synthesis:

Fmoc chemistry was developed by Eric Atherton and Bob Sheppard at the Laboratory of Molecular Biology in Cambridge in the late 1970's and has been reviewed by Chan and [White](http://www.crbdiscovery.com/overview/our-people.php#white) (Fmoc Solid Phase Peptide Synthesis - A Practical Approach. Oxford University Press, 2000).

In Fmoc solid-phase peptide synthesis, the peptide chain is assembled stepwise, one amino acid at a time, while attached to an insoluble resin support. This allows the reaction by-products to be removed at each step by simple washing. Amino acids are protected at their amino terminus by the Fmoc (9-fluorenylmethoxycarbonyl) group and coupled to the growing chain after activation of the carboxylic acid terminus. The Fmoc group is then removed by piperidine treatment and the process repeated. After the peptide has been assembled it is removed from the resin by treatment with trifluoroacetic acid (TFA). At the same time, protecting groups on amino acid side chains are also removed yielding the crude linear peptide. One-step purification by reverse-phase HPLC is often sufficient to obtain the peptide in >95% purity. Fmoc synthesis is mild, flexible and versatile and consequently offers more synthetic options than the alternative Boc chemistry.

**Figure S1** The basic steps in solid phase peptide synthesis using Fmoc chemistry. Fmoc: 9-fluorenylmethoxycarbonyl; AA: Amino Acid; P: protecting groups on amino acid side chains; A: activation;


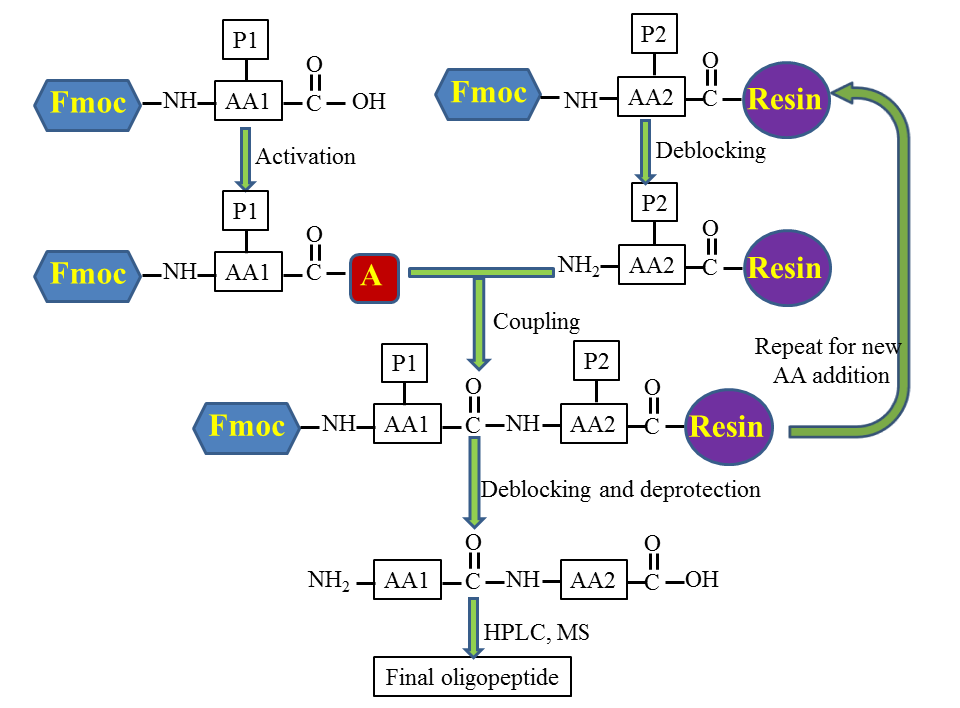

Supplement: Additional file 1: Figure S1 — Fmoc SPPS solid-phase, peptide synthesis. [file 1472-6750-14-32-S1.docx]
